# Phenotypic and Genetic Characterization of Antimicrobial Profiles of *Helicobacter pylori* Strains in Cuba

**DOI:** 10.3329/jhpn.v28i2.4881

**Published:** 2010-04

**Authors:** Rafael Llanes, Claudia Soria, Shigeo Nagashima, Nobumichi Kobayashi, Angela Gala, Daymi Guzmán, Onelkis Feliciano, Lidunka Valdés, Oderay Gutiérrez, Heriberto Fernández, Alina Llop, Akihiro Wada

**Affiliations:** ^1^ Microbiology, Clinical and Epidemiology Branch, Institute Pedro Kouri, Havana, Cuba; ^2^ Department of Hygiene, Sapporo Medical University, Sapporo, Japan; ^3^ Department of Microbiology, Universidad Austral de Chile, Valdivia, Chile; ^4^ Department of Bacteriology, Institute of Tropical Medicine, Nagasaki University, Japan

**Keywords:** Antibiotic resistance, Gene mutations, *Helicobacter pylori*, Cuba

## Abstract

The study evaluated the antibiotic resistance patterns of *Helicobacter pylori* strains against metronidazole and clarithromycin in a hospital in Havana, Cuba. Eighty-five percent, 22.5%, and 10% of 40 *H. pylori* strains investigated were resistant to metronidazole, ciprofloxacin, and clarithromycin respectively but all were susceptible to amoxicillin and tetracycline. RdxA truncation was found only in metronidazole-resistant strains. In such strains, reported are eight and two novel mutations in the *rdxA* and *frxA* genes respectively. Two-point mutations in the *23S rRNA* genes of clarithromycin-resistant strains were detected. A high prevalence of metronidazole resistance was found in Cuban *H. pylori* strains. Mutations in the *rdxA* gene may contribute more significantly than *frxA* gene to the high level of resistance to metronidazole. This study supports the need to continue monitoring the antibiotic susceptibility in *H. pylori* in Cuba to guide the treatment of such infection.

## INTRODUCTION

*Helicobacter pylori* is responsible for chronic gastritis, peptic ulcer disease, and gastric mucosa-associated lymphoid tissue lymphoma and is a major risk factor for the development of gastric adenocarcinoma (1). Eradication of such bacteria by treatment with two antimicrobial agents—clarithromycin (CLA) and amoxicillin (AMX) or metronidazole (MTZ)—and a proton pump inhibitor is recommended by various consensus groups (2).

Investigation on the susceptibility of *H. pylori* to antibiotics is one of the main factors associated with successful eradication therapy (3).

MTZ resistance in *H. pylori* is caused by null mutations in the *rdxA* gene and less frequently by mutations in *frxA* and *fdxB* genes. Resistance to CLA is associated with point mutations in the 23S *rRNA* gene (2).

The prevalence of *H. pylori*-resistant strains is high in naive patients and even higher in patients suffering unsuccessful eradication therapy (4).

In Cuba, the prevalence of *H. pylori*-associated infection among children and adults is 42.4% and 82.7% respectively (5); however, no information is available on antimicrobial susceptibility to commonly-used drugs for the treatment of infection due to *H. pylori*. This study was conducted to evaluate (a) resistance of five antimicrobial agents and (b) genetic basis for MTZ and CLA resistance in *H. pylori* strains from a hospital in Havana city, Cuba. The study also investigated the demographic and clinical factors associated with antibiotic resistance.

## MATERIALS AND METHODS

### Patients, gastric biopsies, and culture

In total, 70 consecutive patients aged 19–68 years were enrolled, and of them, 42 were male and 28 were female. They attended the hospital of the Institute Pedro Kouri, Havana, Cuba, for upper gastrointestinal endoscopy during the last four months of 2005. Of the patients, 51 had non-ulcer dyspepsia (NUD), and 19 had peptic ulcer (PU).

A gastric biopsy specimen obtained from the antrum of each patient was cultured onto selective Columbia agar plates (Oxoid, UK), with 10% sheep-blood and Dent supplement (Oxoid). The plates were incubated at 37 °C in a micro-aerobic atmosphere (Campybag, Oxoid) for 3–5 days. A culture was considered positive if typical *H. pylori* colonies were observed and the microorganisms grown were positive in catalase, oxidase and urease tests (5).

### Susceptibility testing

The minimum inhibitory concentrations (MICs) were determined for MTZ, CLA, AMX, ciprofloxacin (CIP), and tetracycline (TET) by the E-test method (AB Biodisk, Solna, Sweden) as described by the Clinical and Laboratory Standards Institute (CLSI). Mueller Hinton agar (Oxoid), with 5% of aged sheep-blood, was used as culture medium for determining antibiotic susceptibility (6). All tests were performed thrice, and *H. pylori* ATCC 43504 was used as a control strain.

Susceptibility to CLA was interpreted according to the guidelines of the CLSI (6). Since the CLSI has not designated breakpoints for other antimicrobials in *H. pylori*, the following MIC values were used in defining resistance: MTZ ≥8 mg/L, AMX and CIP ≥1 mg/L, and TET ≥2 mg/L (7).

### PCR and DNA sequence analysis

Bacterial genomic DNA was extracted using the DNeasy tissue kit (QIAGEN, Japan). Resistant genes—*rdxA, frxA, and 23S rRNA*—were amplified using PCR with specific primers as previously described (8, 9). Amplified DNA was purified using Wizard-SV gel and PCR Clean-Up System (Promega, USA) and was then sequenced in both directions using the ABI PRISM 3100 automatic sequencer (Applied Biosystems, USA). For identification of ribosomal mutations, sequences were compared with the genomic sequences of *H. pylori* reference strains J99 and 26695 (8). Comparisons were done using the software available over the Internet at the National Center for Biotechnology Information website (http://www.ncbi.nlm.nih.gov), and multiple alignments were performed using the Genetyx-Mac software (version 11.2) (Genetyx Corporation, Japan) (10).

### Statistical analysis

Statistical analysis was carried out with the SPSS software for Windows (version 11.0) (Chicago, USA). The chi-square and Fischer's tests were used for determining the differences between patterns of resistance by age-group, sex, and clinical features. The p values of <0.05 were regarded as statistically significant.

### Ethics

The present study was conducted in accordance with the Declaration of Helsinki and the ethical committee of the Institute Pedro Kouri. Written informed consent was obtained from each subject to participate in the study. The gastric mucosa biopsy for diagnosis of *H.* pylori-associated infection is part of the routine management in patients with dyspepsia and peptic ulcer.

## RESULTS

### Antimicrobial susceptibility

Forty-six (65.7%) of the 70 enrolled patients were positive for *H. pylori* by culture. Of the 46 strains, 40 were available for antimicrobial susceptibility testing with 67.5% (27 of 40) and 32.5% (13 of 40) of the strains from non-ulcer dyspepsia and peptic ulcer patients respectively.

Eighty-five percent, 22.5%, and 10% of the 40 *H. pylori* strains investigated were resistant to MTZ, CIP, and CLA respectively but all were susceptible to AMX and TET. The MIC_50_ and MIC_90_ values for MTZ were 256 mg/L. In the case of CIP, the MIC_50_ was 0.125 mg/L, and the MIC_90_ was 32 mg/L (Table 1).

**Table 1.** Susceptibility of 40 *Helicobacter pylori* Cuban strains to five antibiotics

Antimicrobial	Antimicrobial susceptibility				MIC values (mg/L)		
	Susceptible		Resistant				
	No.	%	No	%	MIC range	MIC_50_	MIC_90_
Amoxicillin	40	100	-		0.016–1	0.016	0.03
Clarithromycin	36	90	4	10	0.016–256	0.016	0.125
Ciprofloxacin	31	77.5	9	22.5	0.016–32	0.125	32
Metronidazole	6	15	34	85	1–256	256	256
Tetracycline	40	100	-	0.016–1	0.06	0.25

MIC=Minimum inhibitory concentration

Our study revealed multiple antibiotic resistance patterns in 25% (10/40) of *H. pylori* strains investigated. Combined resistance to MTZ-CIP (6/10 strains) was most frequently found, followed by patterns—MTZ-CLA and MTZ-CIP-CLA, with two strains each.

Antimicrobial susceptibilities of the strains collected from patients with PU and NUD, males and females and stratified in age-groups below and above 40 years were compared, and no significant difference (p>0.05) in antimicrobial resistance was observed among these groups (Table 2).

**Table 2.** Resistance of 40 *Helicobacter pylori* Cuban strains to three antibiotics by sex, age, and clinical features of patients

Antimicrobial	No. (%) of resistant strains in patients					
	PU	NUD	Male	Female	<40 years	>40 years
	(n=13)	(n=27)	(n=22)	(n=18)	(n=13)	(n=27)
Metronidazole	11 (84.6)	23 (85.2)	21 (95.4)	13 (72.2)	11 (84.6)	23 (85.2)
	p=0.67		p=0.10		p=0.67	
Clarithromycin	1 (7.7)	3 (11.1)	3 (13.6)	1 (5.6)	-	4 (14.8)
	p=0.82		p=0.75		p=0.36	
Ciprofloxacin	2 (15.4)	7 (25.9)	5 (22.7)	4 (22.2)	2 (15.4)	7 (25.9)
	p=0.73		p=0.73		p=0.73	

NUD=Non-ulcer dyspepsia;

PU=Peptic ulcer

### Characterization of *rdxA* and *frxA* genes of MTZ-sensitive and resistant strains

A MTZ-sensitive strain (#56) exhibited amino acid substitution (missense mutation). In the case of MTZ-resistant strains, one had a missense mutation (#191); seven demonstrated amino acid substitutions (#84C, #93, #113, #114, #127, #227, and #230); two had nonsense mutations (#81 and #117); and three others (#67, #84A, and #146) showed both nonsense mutations and amino acid substitutions that resulted in a truncated RdxA protein at positions 35, 75, 139, and 211. One strain (#17) remained unchanged for the *rdxA* gene (Table 3).

**Table 3.** Minimum inhibitory concentration and mutations detected in the *rdxA* and *frxA* genes from metronidazole-sensitive and resistant *Helicobacter pylori* strains

Isolate	MIC (mg/L)	*rdxA* gene		*frxA* gene	
		Change in nucleotide sequence	Change in amino acid sequence	Change in nucleotide sequence	Change in amino acid sequence
56	1.5	-	Ala (37) →Val	-	-
112	0.75	-	-	Frameshift (nt deletion at position 54)	Stop codon at position 39
176	4	-	-	Frameshift (nt deletion at position 54)	Stop codon at position 39
17	256	-	-	Frameshit (nt deletion at position 54)	Stop codon at position 74
			Pro (106)→Ser*	Frameshift (nt deletion at position 54)	Stop codon at position 39**
67	256	-	Val (111)→Ala*		
			Glu (139)→Stop codon*		
81	256	-	Glu (35)→Stop codon	Frameshit (nt deletion at position 54)	Stop codon at position 39**
			Arg (10)→Ile		
84A	256	-	Arg (16)→Cys,	Frameshift (nt deletion at position 54)	Stop codon at position 39**
			Glu (35)→Stop codon		
			Arg (10)→Ile	Frameshift (nt deletion at position 54)	Stop codon at position 39**
84C	256	-	Arg (16)→Cys		Stop codon at position 39**
			Thr (208)→Ala*		
93	128	-	His (97)→Thr	Frameshift (nt deletion at position 54)	Stop codon at position 39**
			Arg (16)→Cys		Thr (110)→Ser,
113	256	-	Ser (81)→Leu	-	Glu (169)→Lys,
			Pro (106)→Ser*	-	Glu (199)→Stop codon*
			Val (111)→Ala*		
114	256	-	Arg (16)→His	-	Glu (169)→Lys
					Met (126)→Phe
117	256	-	Glu (75)→Stop codon	Frameshift (nt deletion at position 98)	Stop codon at position 39**
127	128	-	Asn (14)→Thr*	Frameshift (nt deletion at position 54)	Stop codon at position 39**
146	256	-	Glu (171)→Lys*	Frameshift (nt insertion at position 248)	Stop codon at position 83*
			Leu (211)→Stop codon		
191	128	Missense (deletion of three nt at position 36–38)		Frameshift (nt deletion at position 54)	Stop codon at position 39**
227	256	-	Arg (16)→His	Frameshift (nt deletion at position 54)	Stop codon at position 39**
			Met (56)→Val*		
230	64	-	Arg (16)→Cys,	Frameshift (nt deletion at position 54)	Stop codon at position 39**
		-	His (97)→Tyr		
			Arg (191) →Lys*		

*Mutational pattern not previously reported in *H. pylori* strain resistant to metronidazole;

**This mutational pattern was also observed in *H. pylori* strains susceptible to metronidazole;

MIC=Minimum inhibitory concentration;

nt=Nucleotide

With a few exceptions, the open reading frame for the *frxA* gene was disrupted by a deletion of a nucleotide (nt) at position 54 and 98, and in the resistant strain #146 by a nt insertion at position 248. These events led to the occurrence of a stop codon at positions corresponding to amino acids 39, 74, or 83. In resistant strains #113 and #114, some point mutations were observed (Table 3).

### Analysis of mutations in CLA-resistant and susceptible strains

Of the three resistant strains investigated, two (66.7%) harboured 23S rDNA mutations at position A2142G (n=1), or in both, A2142 and A2143 positions (n=1). These two strains exhibited high-level resistance to CLA (MIC >256 mg/L). The remaining three strains (two susceptible and a CLA-resistant one with an MIC value of 8 mg/L) did not show such alterations at the conserved V domain of genes-encoding *23S rRNA* (data not shown).

## DISCUSSION

In patients undergoing endoscopy, therapy should be tailored based on antimicrobial susceptibility data (11).

Resistance to antimicrobials, as detected in culture, is of particular concern with *H. pylori* as a major cause of eradication failure. Resistance to MTZ is the most common type of resistance in this pathogen (3). The high frequency of resistance to this drug in *H. pylori* strains studied might be due to its frequent use for the treatment of intestinal parasites and gynaecological disorders. MTZ also represents the third most-used antibiotic by Cuban patients attending the primary healthcare system (12).

It is noteworthy that the mutation Ala (37) →Val, detected in a MTZ-susceptible strain, was different from those observed in MTZ-resistant ones, and so could not be essential for resistance. Some mutations detected in the *rdxA* gene from the resistant strains—Arg(10)→Ile; Arg(16)→Cys; Arg(16)→His; His(97)→Thr; Ser(81)→Leu—have been described previously (8, 9, 13).

An interesting finding in MTZ-resistant strains in our study was the detection of several mutations not previously described in the literature in *rdxA* gene: [Glu (139)→Stop codon; Pro(106)→Ser; Val(111)→Ala; Thr(208)→Ala; Asn(14)→Thr; Glu (171)→Lys; Met(56)→Val; Arg(191)→Lys] and in *frxA* gene: [Glu(199)→Stop codon; Stop codon at position 83], which might be associated with resistance.

Some reports established that inactivation of *frxA* without mutations in *rdxA* could not cause MTZ resistance (8, 13). Our results suggest that alterations of *frxA* alone produced resistance, e.g. strain 17. However, as the *frxA* mutations were also observed in MTZ-susceptible strains, we conclude that these are unlikely to contribute to the MTZ resistance of these strains.

CLA is the most powerful antibiotic currently used for the treatment of *H. pylori*. Resistance to this antibiotic considerably reduces the success rate of standard triple therapies (14). The percentage of CLA resistance found in this study and the type of mutations in the *23S rRNA* gene are similar to reports from the USA, Japan, and Bulgaria (2).

In the current investigation, a moderately-resistant CLA strain lacked any mutation in the analyzed region of the *23S rRNA* gene, and the basis for such resistance remains undetermined. It suggests that other undetected *23S rRNA* mutations may be involved in resistance, which has been reported elsewhere (15). As *H. pylori* contains two copies of *23S rRNA,* sometimes it exhibits a heterozygous condition in which one of the genes is mutated and the other remains normal. This genotype confers a CLA-resistant phenotype (15). In addition, a heterogeneous resistance pattern reflecting mixed infections with susceptible and resistant populations to CLA and other antimicrobials has been observed in *H. pylori* isolates (16).

CLA has never been used for the treatment of *H. pylori*-associated infections in Cuba but other macrolides, such as erythromycin and azithromycin, have been widely used in several infections in this country (17). Cross-resistance between CLA and other macrolides have been described before and may account for the observed resistance (14).

Combined resistance to MTZ and CLA may compromise the effectiveness of current triple therapy regimens for *H. pylori*-associated infections (16). Interestingly, our analysis of resistance patterns showed that strains with dual resistance to these drugs were relatively uncommon (10%) in the study population.

After treatment failures to commonly-used anti-*H. pylori* antibiotics, such as MTZ or CLA, quinolone-based triple therapies have been proposed as rescue regimens (2). The prevalence of resistance to such antimicrobials has been determined in only a limited number of studies (7, 18). The mechanism of resistance to fluoroquinolones in *H. pylori* has been shown to be linked to mutations in the so-called quinolone resistance-determining region (QRDR) of the *gyrA* gene (18). In the present study, we did not investigate the mutations associated with such resistance. In future studies, it would be noticeable to perform the sequencing analysis of the QRDR region in fluoroquinolone-resistant strains. The high percentage of CIP resistance in the present study might be a consequence of its frequent use for the management of several infectious diseases in Cuba (12).

*In vitro* resistance to AMX or TET appears to be rare among primary isolates of *H. pylori* (13) as, in our study, several investigations have shown a very marked susceptibility to these two drugs (2, 5, 14). However, it is necessary to mention that subsequent freezing of *H. pylori* strains at −80 °C can result in the loss of AMX-resistant phenotype (19).

The present current study was conducted to gather preliminary data on antimicrobial susceptibility of *H. pylori* strains from a hospital in Havana, Cuba. Consequently, they are not representative of all Cuban population. The participating hospital centre serves only adult patients from a limited area.

In resume, we found a high frequency of MTZ and CIP resistance in *H. pylori* strains for the first time in Cuba. Mutations in the *rdxA* gene may contribute more significantly than *frxA* gene to the high-level MTZ resistance. The present investigation suggests the need for continuous monitoring of the antimicrobial susceptibility in *H. pylori* strains in Cuba.

### Public-health implications

Overall, the weight of evidence suggests that eradication of *H. pylori* will prevent the majority of gastric cancers that are caused by gastric inflammation and many that are associated with gastric atrophy. Screening of population and treatment for infection due to *H. pylori* is an appealing strategy (20) as the aim is to prevent disease and its complications. The data on antimicrobial susceptibilities provided by the present study is critical to guide the clinicians on the effectiveness of treatment regimens.

## ACKNOWLEDGEMENTS

The study is supported, in part, by the Canadian International Development Agency (CIDA) through the Global Health Research Initiative (File: 102172-006).
